# Do knee alignment patterns differ between Middle Eastern and East Asian populations? A propensity-matched analysis using artificial intelligence

**DOI:** 10.1186/s43019-025-00261-w

**Published:** 2025-03-03

**Authors:** Jisoon Park, Oussama Chaar, Jayakrishnan Narayanakurup, Alamedin Sobhe Abdelsamea Abdelhamead, Du Hyun Ro, Sung Eun Kim

**Affiliations:** 1 Department of Orthopedic Surgery, Burjeel Hospital Abu Dhabi, Abu Dhabi, United Arab Emirates; 2 Hip & Knee Center, Mediclinic Airport Road Hospital, Abu Dhabi, United Arab Emirates; 3 Department of Orthopedic Surgery, Seoul National University College of Medicine, Seoul National University Hospital, 101 Daehak-ro, Jongno-gu, Seoul, 110-744 Republic of Korea; 4 CONNECTEVE Co. Ltd., Seoul, South Korea; 5 Innovative Medical Technology Research Institute, Seoul National University Hospital, Seoul, South Korea; 6 Department of Biomedical Informatics, Harvard University, Cambridge, MA USA

**Keywords:** Knee alignment, Osteoarthritis, Propensity score-matched, Middle East, South Korea, Populations

## Abstract

**Introduction:**

Population-based differences in knee alignment patterns may impact osteoarthritis (OA) progression. This study compares lower extremity alignment in knee OA between Middle Eastern (UAE) and East Asian (South Korean) populations using artificial intelligence (AI)-assisted analysis.

**Methods:**

A retrospective review included patients with knee symptoms from South Korea (2009–2019) and the United Arab Emirates (UAE) (2015–2024). Exclusion criteria comprised prior knee surgeries, significant bony attrition, and low-quality radiographs. Propensity score matching controlled for age and sex differences between populations. Alignment parameters (hip–knee–ankle angle (HKA), medial proximal tibial angle (MPTA), lateral distal femoral angle (LDFA), and joint line convergence angle (JLCA)) and OA severity (Kellgren–Lawrence (KL) grade) were measured using artificial intelligence (AI) software, along with the analysis of coronal plane alignment of the knee (CPAK) classification. Subanalyses by sex and age group (under 40, 40–60, and over 60 years) were also conducted.

**Results:**

A total of 1098 UAE and 7138 South Korean patients (2196 and 14,276 knees, respectively) were evaluated in this study. Post-matching (1:2), body mass index was significantly higher in UAE patients (*p* < 0.001). CPAK type 1 was predominant in UAE male patients (42.4%), whereas type 2 was more common in South Korean male patients (30.6%). Female patients in both populations predominantly exhibited CPAK type 2 (UAE 30.6%; South Korea 35.3%). UAE patients showed a lower MPTA with increasing age, indicating a trend toward more varus alignment in older individuals.

**Conclusions:**

A propensity score-matched analysis revealed significant alignment differences between Middle Eastern and East Asian populations, underscoring the importance of population-specific considerations in OA management.

## Introduction

Knee osteoarthritis (OA) affects 16–30% of the global elderly population, with its prevalence steadily increasing worldwide [1]. In the Middle East, the prevalence reaches up to 47.1%, varying by study criteria and demographics [2, 3]. Key radiographic features, such as the medial proximal tibial angle (MPTA) and lateral distal femoral angle (LDFA), are associated with varus deformity, a factor that can accelerate OA progression. Asian populations are reported to exhibit a greater susceptibility to varus deformities than other populations [4, 5].

Understanding lower limb alignment patterns is important for predicting OA progression and planning surgical interventions [6]. Key alignment parameters such as hip-knee-ankle angle (HKA), MPTA, LDFA, and joint line convergence angle (JLCA) provide valuable insights into coronal alignment and OA severity. However, data on alignment patterns in Middle Eastern populations remain limited and are often based on small samples [7, 8], which hampers the development of region-specific treatment protocols. Previous studies on population-based alignment have aimed to standardize measurement methods by adopting the concept of constitutional alignment [9], and categorizing knee phenotypes through the Coronal Plane Alignment of the Knee (CPAK) Classification [10]. Building upon this classification, researchers have sought to clarify global variations in knee alignment [6]. However, direct comparisons of knee alignment between different populations can be flawed, as CPAK types may vary between countries and among healthy and arthritic subjects, influenced by factors such as age and sex [6, 10–12].

In addition, measuring alignment in large population-based cohorts through radiographs is time-intensive and subject to interobserver variability. Advances in artificial intelligence (AI) offer promising solutions to these challenges. AI-assisted analysis has demonstrated greater consistency in OA diagnostics, especially in the automated measurement of alignment parameters and classification of disease severity [13–15]. These software tools have been validated through comparisons with clinicians and gold standard measurements, showing an accuracy on par with orthopedic specialists for key parameters such as HKA, MPTA, LDFA, and JLCA [15]. The use of AI for automated analysis ensures higher inter-rater reliability and significantly reduces the time required for measurement, making it a powerful tool for large-scale population-based studies.

This study aims to conduct a comparative analysis of knee alignment parameters (HKA, MPTA, LDFA, and JLCA), along with CPAK classification, in two populations—the United Arab Emirates (UAE) and South Korea—using AI-based software, with specific attention to age- and sex-based variations. We hypothesize that significant population-specific differences exist in knee alignment parameters.

## Patients and methods

This study was approved by the Institutional Review Board (IRB no. H-2406-148-1547 and BH/REC/063/24). A retrospective review was conducted for patients presenting with knee symptoms in South Korea from January 2009 to December 2019 and in the UAE from March 2015 to August 2024, with data obtained from one tertiary hospital in each country. A total of 8638 patients were initially enrolled in the study. Patients were excluded if they were under 18, had undergone knee-related surgeries, had subluxations or dislocations, or had tibial or femoral bony attrition exceeding 5 mm. Patients lacking height or weight information were also excluded, along with those with poor-quality radiographs (e.g., suboptimal contrast, improper positioning, or shape distortion) and images missing or displaying ambiguous anatomical landmarks (e.g., incomplete coverage from the femur head to the tibial plafond or difficulty pinpointing specific landmarks). After applying the exclusion criteria, 1098 UAE patients and 7138 South Korean patients were included in the study.

Alignment parameters and OA severity were measured using ALI version 1.0.0 and CONNEVO KOA version 1.0.0 (CONNECTEVE Co., Ltd., South Korea), from whole-leg and knee anteroposterior radiographs. The measured parameters included HKA, MPTA, LDFA, and JLCA and the Kellgren–Lawrence (KL) grades. The software demonstrated an intraclass correlation coefficient of 0.999 for HKA measurement and 81% accuracy for KL grade 2–4 classification [14–16]. These models were trained using expert-labeled radiographs and were validated with radiographs from multiple institutions. To assess constitutional knee alignment, the coronal plane alignment of the knee (CPAK) classification was applied, which calculates the arithmetic HKA and joint line obliquity (JLO), as described by Macdessi et al. [10]. Patient demographics, including age, sex, and body mass index (BMI), were also collected.

For each patient, the left and right knees were analyzed separately, resulting in a total of 2196 knees from the UAE and 14,276 knees from South Korea. The detailed patient information is shown in Table 1. Propensity score matching was performed on the basis of age and sex to account for significant baseline differences between the two populations. BMI was not included in the matching process owing to its significant differences between the populations, and because it is considered a factor influenced by ethnic variations. A 1:2 matching ratio was chosen to retain a larger sample of South Korean subjects while reducing statistical discrepancies in age and sex across populations, resulting in a final matched sample of 4392 participants. In addition, subgroup analyses were conducted by sex and age group (below 40, 40–60, and over 60 years) to identify factors contributing to alignment patterns.

**Table 1:** Patient characteristics of the two countries (the United Arab Emirates and South Korea) before and after propensity score matching

	Before matching			Propensity score matched (1:2)	
	UAE (*n* = 2196)	South Korea (*n* = 14,276)	*p*-Value	South Korea (*n *= 4392)	*p*-Value
Age (years)	48.7 ± 14.1	59.9 ± 15.8	< 0.001	49.3 ± 14.0	0.998
Sex (male %)	51.5%	24.8%	< 0.001	51.5%	1.000
Body mass index (kg/m^2^)	31.2 ± 6.3	25.4 ± 3.6	< 0.001	25.1 ± 3.9	< 0.001
Kellgren–Lawrence grade					
0	813 (37.0%)	3389 (23.7%)		1889 (43.0%)	
1	420 (19.1%)	1561 (10.9%)		679 (15.4%)	
2	442 (20.1%)	4216 (29.5%)		1097 (25.0%)	
3	384 (17.5%)	3729 (26.1%)		561 (12.8%)	
4	137 (6.2%)	1380 (9.7%)		166 (3.8%)	
Hip-knee-ankle angle	175.1 ± 4.2	175.9 ± 4.9	< 0.001	177.3 ± 3.9	< 0.001
Medial proximal tibial angle	86.4 ± 2.9	86.9 ± 2.7	< 0.001	86.8 ± 2.6	< 0.001
Lateral distal femoral angle	87.9 ± 2.8	87.6 ± 2.7	< 0.001	87.1 ± 2.6	< 0.001
Joint line convergence angle	2.9 ± 2.3	3.3 ± 3.1	< 0.001	2.4 ± 2.5	< 0.001
Arithmetic HKA	−1.5 ± 4.1	−0.8 ± 3.7	< 0.001	−0.3 ± 3.4	< 0.001
Arithmetic JLO	174.3 ± 3.9	174.5 ± 3.8	0.014	173.9 ± 3.8	0.002
CPAK					
1	775 (35.3%)	3662 (25.7%)		973 (22.2%)	
2	657 (29.9%)	4801 (33.6%)		1709 (38.9%)	
3	302 (13.8%)	2303 (16.1%)		829 (18.9%)	
4	161 (7.3%)	1204 (8.4%)		260 (5.9%)	
5	183 (8.3%)	1393 (9.8%)		364 (8.3%)	
6	87 (4.0%)	686 (4.8%)		195 (4.4%)	
7	21 (1.0%)	89 (0.6%)		19 (0.4%)	
8	5 (0.2%)	87 (0.6%)		25 (0.6%)	
9	5 (0.2%)	50 (0.4%)		18 (0.4%)	

HKA, hip-knee-ankle angle; JLO, joint line obliquity; CPAK, coronal plane alignment of the knee

### Statistical analyses

All statistical analyses were performed using Python 3.10.12. Continuous variables were analyzed using the Student’s *t*-test and categorical variables with the chi-squared test. Propensity score matching was conducted to address age and sex differences between the two countries.

## Results

The results of the 1:2 propensity score matching are presented in Table 1. Post-matching, the distribution of KL grade was more balanced between the UAE and South Korean groups. An interesting finding was that after matching, there was a higher proportion of severe OA (KL grades 3 and 4) in the UAE population compared with the South Korean population. Meanwhile, significant differences in BMI and coronal alignment parameters persisted, with HKA, MPTA, and arithmetic HKA being significantly lower in the UAE (all *p* < 0.05), while LDFA, JLCA, and arithmetic JLO were significantly higher in the UAE population (all *p* < 0.05).

**Fig. 1:**
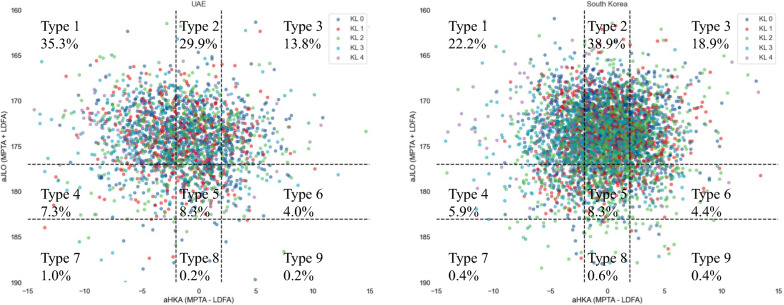
Scatter plots of CPAK classification according to Kellgren–Lawrence grades of the UAE and South Korea

**Fig. 2:**
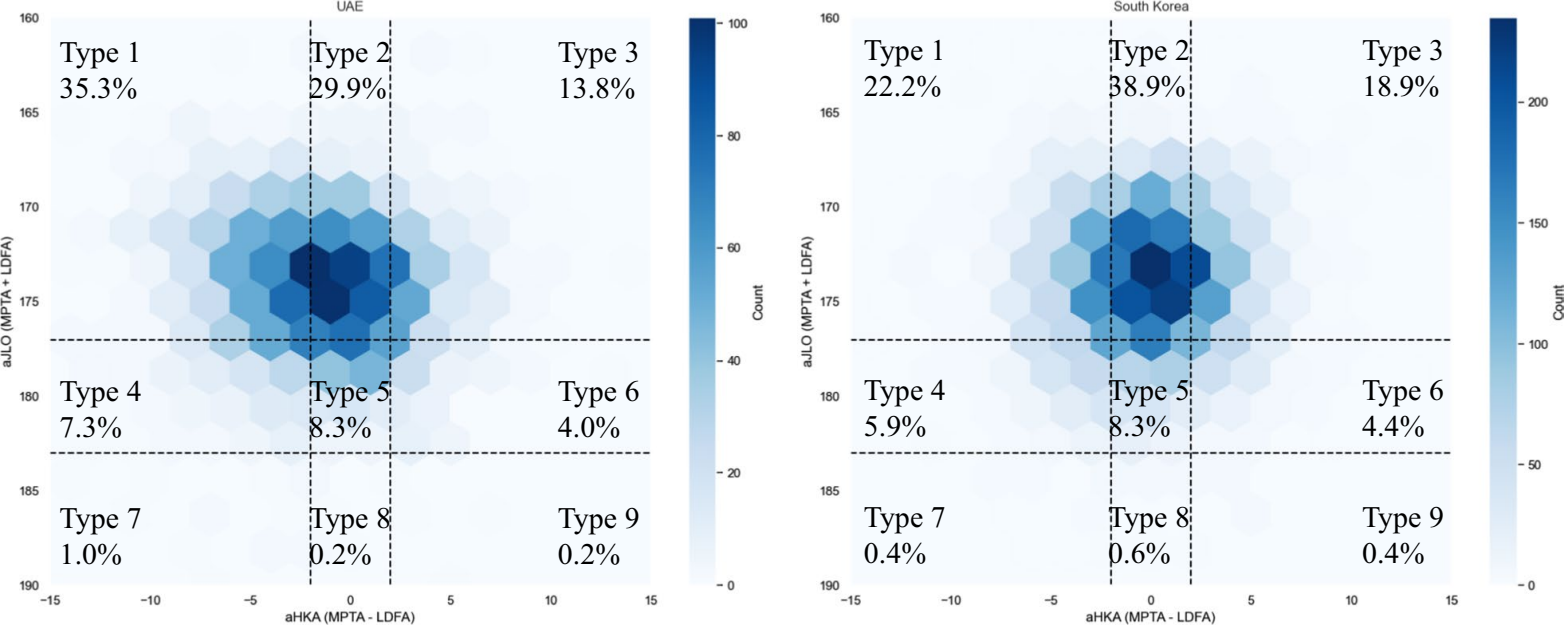
Hexbin plots of CPAK classification of the UAE and South Korea

Analysis of CPAK classifications showed that the most common type in the UAE population was type 1, followed by types 2 and 3. In contrast, the South Korean population predominantly exhibited CPAK type 2, followed by types 1 and 3. This suggests a higher prevalence of varus deformity in the UAE population compared with the South Korean population. For a clearer visualization of the data, Fig. 1 presents scatter plots of CPAK types across both populations. Owing to the density of data points, a hexbin plot (Fig. 2) was generated to enhance clarity. The varying distribution of density is evident, with more densely populated hexbins in CPAK type 1 for the UAE population, while CPAK type 2 predominates in the South Korean population, highlighting the greater prevalence of CPAK type 1 in the UAE and CPAK type 2 in South Korea.

When comparing the populations by sex, significant differences in BMI were observed in both male and female patients (Table 2), with the UAE population having higher BMI values compared with the South Korean population. This difference in BMI was not sex-specific and reflects a general population-level variation. For the radiographic indices, MPTA was significantly lower in the UAE male population (175.2) than in the South Korean population (177.4) (*p* < 0.001). However, among female patients, no significant difference was found in MPTA (*p* = 0.053). For JLCA, no significant difference was observed between the two countries in male patients (*p* = 0.254), while in female patients, JLCA was higher in the UAE (3.6) compared with South Korea (2.5) (*p* < 0.001). In terms of CPAK classifications, UAE male patients predominantly exhibited CPAK type 1, followed by types 2 and 3. In contrast, South Korean male patients predominantly exhibited CPAK type 2, followed by types 1 and 3. This difference in CPAK classification was specific to male patients. For female patients, both populations predominantly exhibited CPAK type 2, with South Korean female patients showing a more balanced distribution of CPAK types 1 and 3.

**Table 2:** Comparison between male and female patients between the United Arab Emirates and South Korea

	Males			Females		
	UAE (*n* = 1130)	South Korea (*n* = 2262)	*p*-Value	UAE (*n* = 1066)	South Korea (*n* = 2130)	*p*-Value
Age	46.8 ± 15.1	47.5 ± 15.1	0.196	50.6 ± 12.7	51.2 ± 12.5	0.238
Body mass index (kg/m^2^)	31.2 ± 6.2	25.5 ± 3.4	< 0.001	31.1 ± 6.3	24.6 ± 4.2	< 0.001
Kellgren–Lawrence grade						
0	487 (43.1%)	1188 (52.5%)		326 (30.6%)	701 (32.9%)	
1	234 (20.7%)	363 (16.0%)		186 (17.5%)	316 (14.8%)	
2	191 (16.9%)	449 (19.8%)		251 (23.6%)	648 (30.4%)	
3	164 (14.5%)	204 (9.0%)		220 (20.7%)	357 (16.8%)	
4	55 (4.9%)	58 (2.6%)		82 (7.7%)	108 (5.1%)	
Hip-knee-ankle angle	175.2 ± 3.9	177.4 ± 3.5	< 0.001	174.9 ± 4.4	177.2 ± 4.4	< 0.001
Medial proximal tibial angle	85.8 ± 2.8	86.5 ± 2.5	< 0.001	87.0 ± 2.8	87.2 ± 2.6	0.053
Lateral distal femoral angle	87.9 ± 2.5	86.9 ± 2.3	< 0.001	87.9 ± 3.1	87.3 ± 2.8	< 0.001
Joint line convergence angle	2.3 ± 1.8	2.2 ± 2.0	0.254	3.6 ± 2.5	2.5 ± 2.9	< 0.001
Arithmetic HKA	−2.1 ± 4.1	−0.4 ± 3.3	< 0.001	−0.9 ± 4.1	−0.2 ± 3.6	< 0.001
Arithmetic JLO	173.7 ± 3.5	173.4 ± 3.5	0.016	174.8 ± 4.1	174.5 ± 4.1	0.034
CPAK						
1	480 (42.4%)	551 (24.4%)		295 (27.7%)	422 (19.8%)	
2	331 (29.3%)	958 (42.4%)		326 (30.6%)	751 (35.3%)	
3	131 (11.6%)	416 (18.4%)		171 (16.1%)	413 (19.4%)	
4	74 (6.5%)	105 (4.6%)		87 (8.2%)	155 (7.3%)	
5	77 (6.8%)	142 (6.3%)		106 (10.0%)	222 (10.4%)	
6	31 (2.7%)	80 (3.5%)		56 (5.3%)	115 (5.4%)	
7	4 (0.4%)	2 (0.1%)		17 (1.6%)	17 (0.8%)	
8	1 (0.1%)	5 (0.2%)		4 (0.4%)	20 (0.9%)	
9	2 (0.2%)	3 (0.1%)		3 (0.3%)	15 (0.7%)	

HKA, hip-knee-ankle angle; JLO, joint line obliquity; CPAK, coronal plane alignment of the knee

**Fig. 3:**
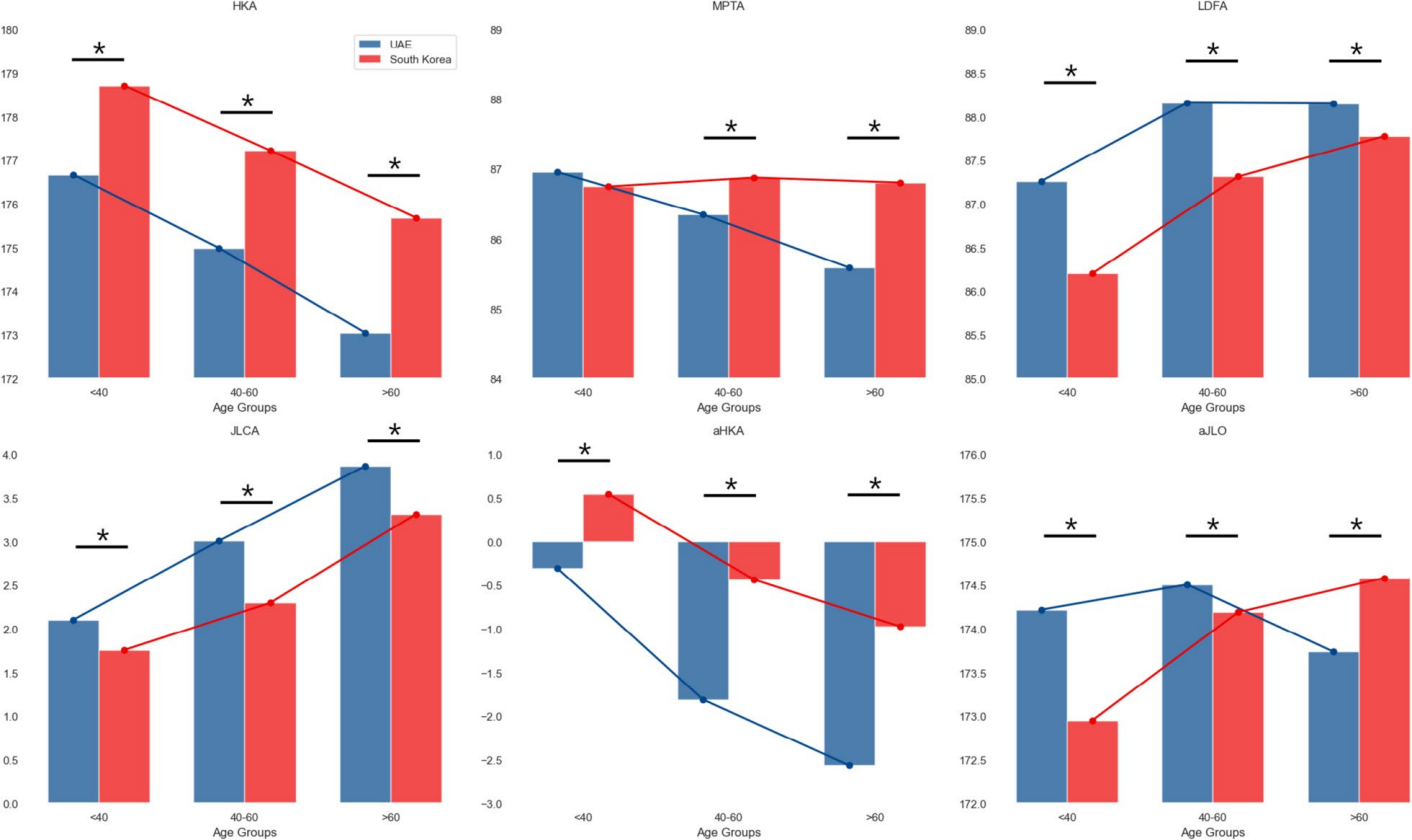
Comparison between age groups between the United Arab Emirates and South Korea; *statistically significant at *p* < 0.05; HKA, hip-knee-ankle angle; MPTA, medial proximal tibial angle; LDFA, lateral distal femoral angle; JLCA, joint line convergence angle; aHKA, arithmetic HKA; aJLO, arithmetic joint line obliquity

Comparisons according to age groups were also performed (Table 3, Fig. 3). Across all age groups, the HKA was lower (more varus) in UAE patients compared with South Korean patients (all *p* < 0.05), with a decreasing HKA trend observed with age. The MPTA was lower in UAE patients over 40 years, showing a trend toward tibia vara with aging. While LDFA exhibited marked differences under age 40 years, the disparity between groups decreased in older age groups. JLCA increased with age in both populations, while the UAE population had higher JLCA. Arithmetic HKA mirrored HKA trends, while arithmetic JLO increased with age in the South Korean population, reversing earlier trends observed in the UAE population.

**Table 3:** Comparison between age groups between the United Arab Emirates and South Korea

	Age group (years)	UAE	South Korea	*p*-value
Hip-knee-ankle angle	Below 40	176.7 ± 2.7	178.7 ± 2.8	< 0.001
	40–60	175.0 ± 4.0	177.2 ± 3.8	< 0.001
	Over 60	173.0 ± 5.1	175.7 ± 4.8	< 0.001
Medial proximal tibial angle	Below 40	87.0 ± 2.8	86.7 ± 2.4	0.088
	40–60	86.3 ± 2.7	86.9 ± 2.6	< 0.001
	Over 60	85.6 ± 3.1	86.8 ± 2.8	< 0.001
Lateral distal femoral angle	Below 40	87.3 ± 2.7	86.2 ± 2.4	< 0.001
	40–60	88.2 ± 2.8	87.3 ± 2.6	< 0.001
	Over 60	88.2 ± 2.8	87.8 ± 2.5	0.011
Joint line convergence angle	Below 40	2.1 ± 1.5	1.8 ± 1.8	< 0.001
	40–60	3.0 ± 2.3	2.3 ± 2.4	< 0.001
	Over 60	3.9 ± 2.7	3.3 ± 2.9	< 0.001
Arithmetic HKA	Below 40	−0.3 ± 3.9	0.5 ± 3.2	< 0.001
	40–60	−1.8 ± 3.8	−0.4 ± 3.3	< 0.001
	Over 60	−2.6 ± 4.6	−1.0 ± 3.8	< 0.001
Arithmetic JLO	Below 40	174.2 ± 4.0	173.0 ± 3.6	< 0.001
	40–60	174.5 ± 3.9	174.2 ± 3.9	0.030
	Over 60	173.7 ± 3.6	174.6 ± 3.7	< 0.001

HKA, hip-knee-ankle angle; JLO, joint line obliquity

## Discussion

This study provides a comparative analysis of lower extremity alignment patterns in knee OA between Middle Eastern (UAE) and East Asian (South Korean) populations. To our knowledge, this is the first study to apply propensity score matching to minimize demographic discrepancies. Our methodology addresses a key limitation of previous CPAK distribution studies that relied primarily on descriptive analyses without demographic adjustments, which enhances the validity of our findings regarding regional differences in alignment patterns. Specifically, our study builds upon the work of Pagan et al., who reported variations in the most common CPAK types across seven countries, with types 1 and 2 being the most prevalent [6]. However, their study lacked adjustment for sex differences, a limitation that we have overcome. Our findings confirm that specific CPAK distributions differ between the UAE and South Korean populations and underscore the importance of adjusting for demographic variables. These results demonstrate that regional factors, in combination with demographic adjustments, are crucial for understanding global variations in knee alignment and OA severity.

An interesting finding of this study was the significant difference in BMI between UAE and South Korean cohorts, even after matching for age and sex. This discrepancy may suggest that BMI may contribute to population-specific alignment variations, as increased joint loading associated with higher BMI may exacerbate joint deformities. Among studies on BMI’s influence on knee alignment, Messier et al. found that a higher BMI was associated with increased knee joint loading and varus alignment which in turn exerted greater knee adduction moments and may contribute to OA progression [17]. These observations support the hypothesis that BMI may play a role in joint loading differences, potentially affecting alignment in populations with higher BMIs.

Our results also showed a predominance of CPAK type 1 among UAE male patients, in contrast to CPAK type 2 in South Korean male patients, indicating anatomical differences between populations. This finding was primarily owing to pronounced arithmetic HKA differences reflecting a smaller MPTA and a higher tendency toward tibia vara deformity in UAE male patients. Among female patients, UAE participants showed a similar, albeit less pronounced, varus alignment tendency in arithmetic HKA, with MPTA nearing statistical significance (*p* = 0.053). This discrepancy may account for the relatively high percentage of advanced OA (KL grades 3 and 4) observed in the UAE population, as smaller MPTA increases medial compartment load, potentially accelerating OA progression [18, 19]. Combined with the higher BMI in UAE patients, these findings align with prior studies indicating a higher prevalence of varus deformity and OA in Middle Eastern populations [2, 3].

Female participants from both populations demonstrated a predominant CPAK type 2 distribution, a pattern distinct from that of their male counterparts. Similar BMI levels between UAE male and female patients (31.2 versus 31.1) suggest that biological sex-related factors may influence knee alignment independently of BMI, especially in the UAE population. Studies have shown sex-specific differences in coronal alignment; for example, Hovinga et al. found that Japanese and Caucasian women exhibited more valgus alignment compared with their male counterparts [20]. Another study found that men displayed greater varus alignment in early or no OA stages, whereas women exhibited higher valgus alignment at advanced OA stages [21]. These findings, in conjunction with our results, suggest that sex differences are crucial considerations in surgical planning. In addition, our sex-stratified analysis revealed no significant differences in MPTA among female participants or JLCA among male participants across the two countries. Given that MPTA is influenced by tibial structure and JLCA by surrounding knee soft tissues, this may imply that male knee alignment is more affected by bony structures, while female alignment may be influenced by soft tissue adaptations.

The age-stratified analysis showed that varus alignment was more prominent across all age groups in the UAE cohort, with the trend becoming more pronounced with advancing age. The lower MPTA in UAE subjects over 40 suggests progressive tibia vara with aging, possibly reflecting cumulative effects of regional biomechanical or lifestyle factors on knee alignment [22, 23]. The similarities in age-related alignment differences between the two groups suggest that skeletal changes with age may be a universal phenomenon, potentially occurring across diverse populations [24]. This observation aligns with a study by Colyn et al., which found that MPTA and JLCA increased as OA progressed, consistent with our findings [25].

These results imply that lifestyle factors beyond genetic predisposition may play a role in shaping skeletal structures over time. For example, traditional sitting postures in the Middle East, where kneeling or sitting on the floor is common, could contribute to varus alignment in the lower extremities over time [22, 23]. This observation is similar to findings in East Asian populations, where floor-sitting habits have been associated with distinct alignment characteristics, suggesting that prolonged lifestyle habits can significantly impact skeletal development [26, 27].

Moreover, these findings emphasize the need for comparative studies across diverse regions and lifestyles, such as Western societies, where sitting on chairs is the norm, and physical activity and sports are culturally prominent. A broader understanding of how various lifestyles influence alignment patterns would provide a more comprehensive view of skeletal adaptations worldwide. Ultimately, examining the link between lifestyle and skeletal alignment could provide critical insights for developing region-specific prevention and treatment strategies, potentially offering valuable guidance for personalized care. These insights may contribute to identifying common yet diverse skeletal adaptation patterns, thereby supporting the development of tailored approaches in knee OA care.

## Limitations

This study is not without limitations. As a retrospective analysis, it cannot establish causality between regional factors and alignment patterns in knee OA. Prospective studies with longitudinal follow-ups are required to validate these findings and to investigate the underlying mechanisms influencing alignment patterns across different populations. In addition, this study did not account for the specific ethnic backgrounds within the populations, which may also affect alignment patterns [28]. Another limitation is that the patients’ radiographs may have been affected by positioning. However, a study by Chung et al. found that HKA was influenced only by flexion contracture, while MPTA and LDFA remained unaffected. Given that CPAK is based on MPTA and LDFA, we selected this method as a reliable measure for evaluating alignment in this study [29].

## Conclusions

Significant differences in lower extremity alignment patterns exists between Middle Eastern and East Asian populations, emphasizing the importance of population-specific considerations in OA management.

## Data Availability

Data available on request from the authors.
